# COVID-19 mortality may be reduced among fully vaccinated solid organ transplant recipients

**DOI:** 10.1371/journal.pone.0279222

**Published:** 2022-12-21

**Authors:** Micaela Sandoval, Duc T. Nguyen, Howard J. Huang, Stephanie G. Yi, R. Mark Ghobrial, A. Osama Gaber, Edward A. Graviss

**Affiliations:** 1 Department of Pathology and Genomic Medicine, Houston Methodist Research Institute, Houston, TX, United States of America; 2 Department of Epidemiology, Human Genetics & Environmental Sciences, The University of Texas Health Science Center School of Public Health, Houston, TX, United States of America; 3 J.C. Walter Jr Transplant Center, Weill Cornell Medical College, Houston Methodist Hospital, Houston, TX, United States of America; 4 Department of Medicine, Houston Methodist Hospital, Houston, TX, United States of America; 5 Department of Surgery, Houston Methodist Hospital, Houston, TX, United States of America; Imperial College Healthcare NHS Trust, UNITED KINGDOM

## Abstract

**Background:**

Solid organ transplant (SOT) recipients are at increased risk for morbidity and mortality from COVID-19 due to their immunosuppressed state and reduced immunogenicity from COVID-19 mRNA vaccines. This investigation examined the association between COVID-19 mRNA vaccination status and mortality among SOT recipients diagnosed with COVID-19.

**Methods & findings:**

A retrospective, registry-based chart review was conducted investigating COVID-19 mortality among immunosuppressed solid organ transplant (SOT) recipients in a large metropolitan healthcare system in Houston, Texas, USA. Electronic health record data was collected from consecutive SOT recipients who received a diagnostic SARS-CoV-2 test between March 1, 2020, and October 1, 2021. The primary exposure was COVID-19 vaccination status at time of COVID-19 diagnosis. Patients were considered ‘fully vaccinated’ at fourteen days after completing their vaccine course. COVID-19 mortality within 60 days and intensive care unit admission within 30 days were primary and secondary endpoints, respectively. Among 646 SOT recipients who were diagnosed with COVID-19 at Houston Methodist Hospital between March 2020, and October 2021, 70 (10.8%) expired from COVID-19 within 60 days. Transplanted organs included 63 (9.8%) heart, 355 (55.0%) kidney, 108 (16.7%) liver, 70 (10.8%) lung, and 50 (7.7%) multi-organ. Increasing age was a risk factor for COVID-19 mortality, while vaccination within 180 days of COVID-19 diagnosis was protective in Cox proportional hazard models with hazard ratio 1.04 (95% CI: 1.01–1.06) and 0.31 (0.11–0.90), respectively). These findings were confirmed in the propensity score matched cohort between vaccinated and unvaccinated patients.

**Conclusions:**

This investigation found COVID-19 mortality may be significantly reduced among immunosuppressed SOT recipients within 6 months following vaccination. These findings can inform vaccination policies targeting immunosuppressed populations worldwide.

## Introduction

Solid organ transplant (SOT) recipients are at increased risk for morbidity and mortality from infectious diseases, including COVID-19, due to their immunosuppressed status and significant comorbidities [1,2]. At the end of 2020, the BNT162b2 (Pfizer-BioNTech) and the mRNA-1273 (Moderna) vaccines became available for high-risk populations, including transplant patients, and transplant centers across the United States instituted vaccination programs. However, recent studies have demonstrated poor humoral and cellular responses among vaccinated SOT recipients compared to patients on transplant waitlists and the general population, prompting concern for poor clinical outcomes of SOT recipients exposed to the severe acute respiratory syndrome coronavirus 2 (SARS-CoV-2) virus post-vaccination [3–20]. This investigation evaluated mortality among SOT recipients diagnosed with COVID-19 before and after implementation of an intensive vaccination program at a large, urban transplant center in Houston, Texas, USA.

## Methods

The study population consisted of all consecutive SOT recipients diagnosed with COVID-19 between March 1, 2020, and October 1, 2021 at Houston Methodist, Houston Texas, USA. SARS-CoV-2 testing was performed as part of the routine screening on SOT recipients. Patients were followed up for outcomes up to December 1, 2021. The Houston Methodist J.C. Walter Jr. Transplant Center is located within the large urban, Texas Medical Center and maintains an active transplant and follow-up program including liver, heart, lung, pancreas, kidney, and multi-organ transplants. COVID-19 mRNA vaccines became available to high-risk populations in the study setting in December 2020, and vaccination programs utilizing the NT162b2 (Pfizer-BioNTech) or the mRNA-1273 (Moderna) vaccines for transplant patients began in January 2021. Demographic and clinical data were retrieved from the Houston Methodist COVID-19 Surveillance and Outcomes Registry (CURATOR), a COVID-19 specific electronic health records (EHR) data mining, surveillance and collection project [21]. Vaccination records and medication data were abstracted directly from the EHR. The Charlson Comorbidity Index was calculated from medical history components as a measure of overall comorbidity burden [22]. SOT recipients were included if they received a positive diagnostic result from a SARS-CoV-2 RNA polymerase chain reaction (PCR) assay or a viral antigen assay at Houston Methodist while undergoing immunosuppressive treatment.

Patients were considered ‘fully vaccinated’ in this study if they had received the second dose of either the NT162b2 or the mRNA-1273 vaccines at least 14 days prior to their first positive SARS-CoV-2 diagnostic test, according to CDC definitions [23]. Patients who tested positive for COVID-19 before the completion of the vaccination course were considered ‘unvaccinated’. Demographic and clinical data were reported as frequencies and proportions for categorical variables and as median and interquartile range (IQR) for continuous variables. Differences in covariates across vaccination status groups were evaluated using the chi-square or Fisher’s exact tests for categorical variables and Kruskal Wallis test for continuous variables, as appropriate. Timepoints for primary and secondary outcome analyses were determined from the distribution of time-to-event data. Cox proportional hazards modeling was performed to determine the characteristics associated with COVID-19 mortality within 60 days. Multivariable logistic regression modeling was utilized to determine the characteristics associated with an ICU admission within 30 days. Variables for the multivariable models were selected on the basis of potential clinical relevance and using the Bayesian Information Criterion. Only baseline characteristics were assessed in regression analyses, as eligibility for specific COVID-19 treatments, including remdesivir and monoclonal antibodies depended on additional factors, such as disease severity upon presentation and time from symptom onset [24,25]. Given the vaccine effectiveness drop after 6 months [26,27], we classified the vaccination status as follow: vaccination status was presented as ‘vaccinated <180 days before COVID-19 diagnosis’, ‘vaccinated 180+ days before COVID-19 diagnosis’, ‘unvaccinated, diagnosed with COVID-19 in 2020’, or ‘unvaccinated, diagnosed with COVID-19 in 2021.’ Unvaccinated patients were categorized by whether they were diagnosed with COVID-19 in 2020 or 2021 as a proxy for advances in COVID-19 treatments which could have influenced survivorship. We conducted a non-replace propensity score (PS) matching (ratio 1:1, caliper 1) between patients with and without vaccination. The matching criteria included age, race, Charlson Comorbidity Index (CCI), year of transplantation, COVID-19 treatments administered at diagnostic encounter (remdesivir, monoclonal antibodies, azithromycin, methylprednisolone, ribavirin, tocilizumab, dexamethasone), immunosuppressant treatments at COVID-19 diagnosis (antithymocyte globulin, tacrolimus, cyclosporine, mycophenolate, azathioprine, sirolimus, everolimus, belatacept, prednisone). Balance of the matching criteria between groups was evaluated using standardized bias percent. Multivariable analysis was also conducted on the PS cohort for mortality with 60 days and ICU admission within 30 days. All analyses were performed on Stata MP version 17.0 (StataCorp LLC, College Station, TX, USA). This retrospective registry-based study was approved by the Houston Methodist institutional review board (PRO00025320) and granted a waiver of informed consent.

## Results

In total, 646 SOT recipients were diagnosed with COVID-19 at Houston Methodist from March 1, 2020 to October 1, 2021. SARS-CoV-2 -positive SOT recipients had a median age of 58 years (47–66 IQR); 357 (55%) were male, 251 (39%) were non-Hispanic White, 161 (25%) non-Hispanic Black, 30 (5%) non-Hispanic Asian, 193 (30%) Hispanic, and 11 (2%) classified as other non-Hispanic race (Table 1). This cohort of SOT recipients had significant comorbidities prior to COVID-19 diagnosis; median score on the Charlson Comorbidity Index was 8 (IQR 5–11), and almost all patients had history of renal disease or diabetes. In total, 315 (57%) SOT patients diagnosed with COVID-19 were kidney recipients, 92 (16%) liver recipients, 59 (11%) lung recipients, 56 (10%) heart recipients, and 36 (6%) multi-organ recipients. Median time from the latest transplant to date of first positive SARS-CoV-2 test was 4.6 years (1.4–9.2 IQR). Within the population of patients undergoing immunosuppressant therapy, 574 (89%) were receiving tacrolimus, 539 (83%) were receiving mycophenolate, and 561 (87%) were receiving prednisone at time of their COVID-19 diagnosis.

**Table 1:** Demographics and clinical characteristics of solid organ transplant recipients diagnosed with COVID-19 by vaccination status.

	Total	Not vaccinated	Vaccinated	
Characteristics	(N = 646)	(n = 505)	(n = 141)	p-value
**Demographics**				
Age at encounter (years), median (IQR)	58 (47–66)	57 (46–65)	61 (52–68)	0.003
Gender				0.77
Female	289 (44.7%)	224 (44.4%)	65 (46.1%)	
Male	357 (55.3%)	281 (55.6%)	76 (53.9%)	
Race/Ethnicity				0.66
Non-Hispanic White	251 (38.9%)	195 (38.6%)	56 (39.7%)	
Non-Hispanic Black	161 (24.9%)	121 (24.0%)	40 (28.4%)	
Non-Hispanic Asian	30 (4.6%)	21 (4.2%)	9 (6.4%)	
Non-Hispanic Hawaiian/Pacific	2 (0.3%)	2 (0.4%)	0 (0.0%)	
Non-Hispanic Native American	4 (0.6%)	4 (0.8%)	0 (0.0%)	
Non-Hispanic Other Race	2 (0.3%)	2 (0.4%)	0 (0.0%)	
Hispanic or Latino	193 (29.9%)	157 (31.1%)	36 (25.5%)	
Unknown	3 (0.5%)	3 (0.6%)	0 (0.0%)	
**Clinical characteristics**				
Body mass index, median (IQR)	28.0 (24.3–32.4)	28.0 (24.3–32.2)	27.8 (24.4–33.0)	0.51
Charlson Comorbidity Index Score, median (IQR)	8 (5–11)	8 (5–11)	8 (6–11)	0.046
Medical history				
Chronic obstructive pulmonary disease	247 (38.2%)	186 (36.8%)	61 (43.3%)	0.17
Tuberculosis	22 (3.3%)	15 (3.0%)	7 (5.0%)	0.29
Dementia	22 (3.4%)	20 (4.0%)	2 (1.4%)	0.19
Myocardial Infarction	226 (35.0%)	169 (33.5%)	57 (40.4%)	0.13
Peripheral vascular disease	329 (50.9%)	232 (45.9%)	69 (48.9%)	0.57
Congestive heart failure	301 (46.6%)	255 (50.5%)	74 (52.5%)	0.7
Cerebrovascular disease	240 (37.2%)	186 (36.8%)	54 (38.3%)	0.77
Diabetes	466 (72.1%)	363 (71.9%)	103 (73.0%)	0.83
Peptic ulcer disease	58 (9.0%)	42 (8.3%)	16 (11.3%)	0.32
Liver disease	269 (41.6%)	209 (41.4%)	60 (42.6%)	0.85
Renal disease	620 (96.0%)	485 (96.0%)	135 (95.7%)	0.81
Hemiplegia	20 (3.1%)	19 (3.8%)	1 (0.7%)	0.09
Cancer	123 (19.0%)	89 (17.6%)	34 (24.1%)	0.09
HIV/AIDS	3 (0.5%)	2 (0.4%)	1 (0.7%)	0.52
**Transplant characteristics**				
Transplanted organ				0.62
Multi-organ	19 (2.9%)	13 (2.6%)	6 (4.3%)	
Heart	63 (9.8%)	53 (10.5%)	10 (7.1%)	
Kidney	355 (55.0%)	274 (54.3%)	81 (57.4%)	
Liver	108 (16.7%)	86 (17.0%)	22 (15.6%)	
Lung	70 (10.8%)	53 (10.5%)	17 (12.1%)	
Kidney/Pancreas	31 (4.8%)	26 (5.1%)	5 (3.5%)	
Year of most recent transplant				0.03
<2015	254 (39.3%)	200 (39.6%)	54 (38.3%)	
2015	47 (7.3%)	38 (7.5%)	9 (6.4%)	
2016	43 (6.7%)	34 (6.7%)	9 (6.4%)	
2017	56 (8.7%)	44 (8.7%)	12 (8.5%)	
2018	49 (7.6%)	41 (8.1%)	8 (5.7%)	
2019	87 (13.5%)	76 (15.0%)	11 (7.8%)	
2020	88 (13.6%)	58 (11.5%)	30 (21.3%)	
2021	22 (3.4%)	14 (2.8%)	8 (5.7%)	
Time from transplant to COVID-19 diagnosis (years), median IQR	4.6 (1.4–9.2)	4.6 (1.4–9.2)	4.8 (1.3–9.5)	0.62
Immunosuppressant treatments at COVID-19 diagnosis				
Antithymocyte globulin	21 (3.3%)	20 (4.0%)	1 (0.7%)	0.06
Tacrolimus	574 (88.9%)	451 (89.3%)	123 (87.2%)	0.49
Cyclosporine	39 (6.0%)	30 (5.9%)	9 (6.4%)	0.85
Mycophenolate	539 (83.4%)	422 (83.6%)	117 (83.0%)	0.87
Azathioprine	14 (2.2%)	13 (2.6%)	1 (0.7%)	0.32
Sirolimus	61 (9.4%)	49 (9.7%)	12 (8.5%)	0.67
Everolimus	14 (2.2%)	9 (1.8%)	5 (3.5%)	0.20
Belatacept	14 (2.2%)	12 (2.4%)	2 (1.4%)	0.75
Prednisone	561 (86.8%)	434 (85.9%)	127 (90.1%)	0.20
**COVID-19 characteristics**				
Date of COVID-19 diagnosis (quarter)				<0.001
Q1 2020	4 (0.6%)	4 (0.8%)	0 (0.0%)	
Q2 2020	68 (10.5%)	68 (13.5%)	0 (0.0%)	
Q3 2020	106 (16.4%)	106 (21.0%)	0 (0.0%)	
Q4 2020	122 (18.9%)	122 (24.2%)	0 (0.0%)	
Q1 2021	138 (21.4%)	131 (25.9%)	7 (5.0%)	
Q2 2021	44 (6.8%)	22 (4.4%)	22 (15.6%)	
Q3 2021	164 (25.4%)	52 (10.3%)	112 (79.4%)	
COVID-19 treatments administered at diagnostic encounter				
Azithromycin	154 (23.8%)	129 (25.5%)	25 (17.7%)	0.05
Methylprednisolone	130 (20.1%)	102 (20.2%)	28 (19.9%)	0.93
Ribavirin	6 (0.9%)	6 (1.2%)	0 (0.0%)	0.35
Tocilizumab	38 (5.9%)	37 (7.3%)	1 (0.7%)	0.002
Dexamethasone	225 (34.8%)	176 (34.9%)	49 (34.8%)	0.98
Remdesivir	231 (35.8%)	169 (33.5%)	62 (44.0%)	0.02
Monoclonal antibodies	136 (21.1%)	76 (15.0%)	60 (42.6%)	<0.001
**Clinical outcomes**				
Total mortality				0.01
Alive at study completion	557 (86.2%)	426 (84.4%)	131 (92.9%)	
Expired	89 (13.8%)	79 (15.6%)	10 (7.1%)	
Cause of death (n = 89)				0.85
Graft rejection/failure	6 (6.7%)	6 (8%)	0 (0%)	
Cardiac arrest	3 (3.4%)	3 (4%)	0 (0%)	
Respiratory failure	3 (3.4%)	3 (4%)	0 (0%)	
Multi-organ failure	1 (1.1%)	1 (1%)	0 (0%)	
Sepsis	2 (2.3%)	2 (3%)	0 (0%)	
COVID-19	70 (78.7%)	61 (77%)	9 (90.0%)	
Myocardial infarction	1 (1.2%)	1 (1%)	0 (0%)	
Other	3 (3.4%)	2 (3%)	1 (10.0%)	
Patient status 30 days post COVID-19 diagnosis				
Ever hospitalized	473 (73.2%)	384 (76.0%)	89 (63.1%)	0.004
Ever admitted to ICU	136 (21.1%)	117 (23.2%)	19 (13.5%)	0.01
Expired	56 (8.7%)	47 (9.3%)	9 (6.4%)	0.31
Cause of death within 30 days of diagnosis (n = 56)				0.30
COVID-19	54 (96%)	46 (98%)	8 (89%)	
Other	2 (4%)	1 (2%)	1 (11%)	

Values are in number (%) unless otherwise specified; IQR: *Interquartile range*; ICU: *Intensive care unit;* Differences between exposure groups were compared using the chi-square or Fisher’s exact tests for categorical variables and Kruskal Wallis test for continuous variables.

**Fig 1:**
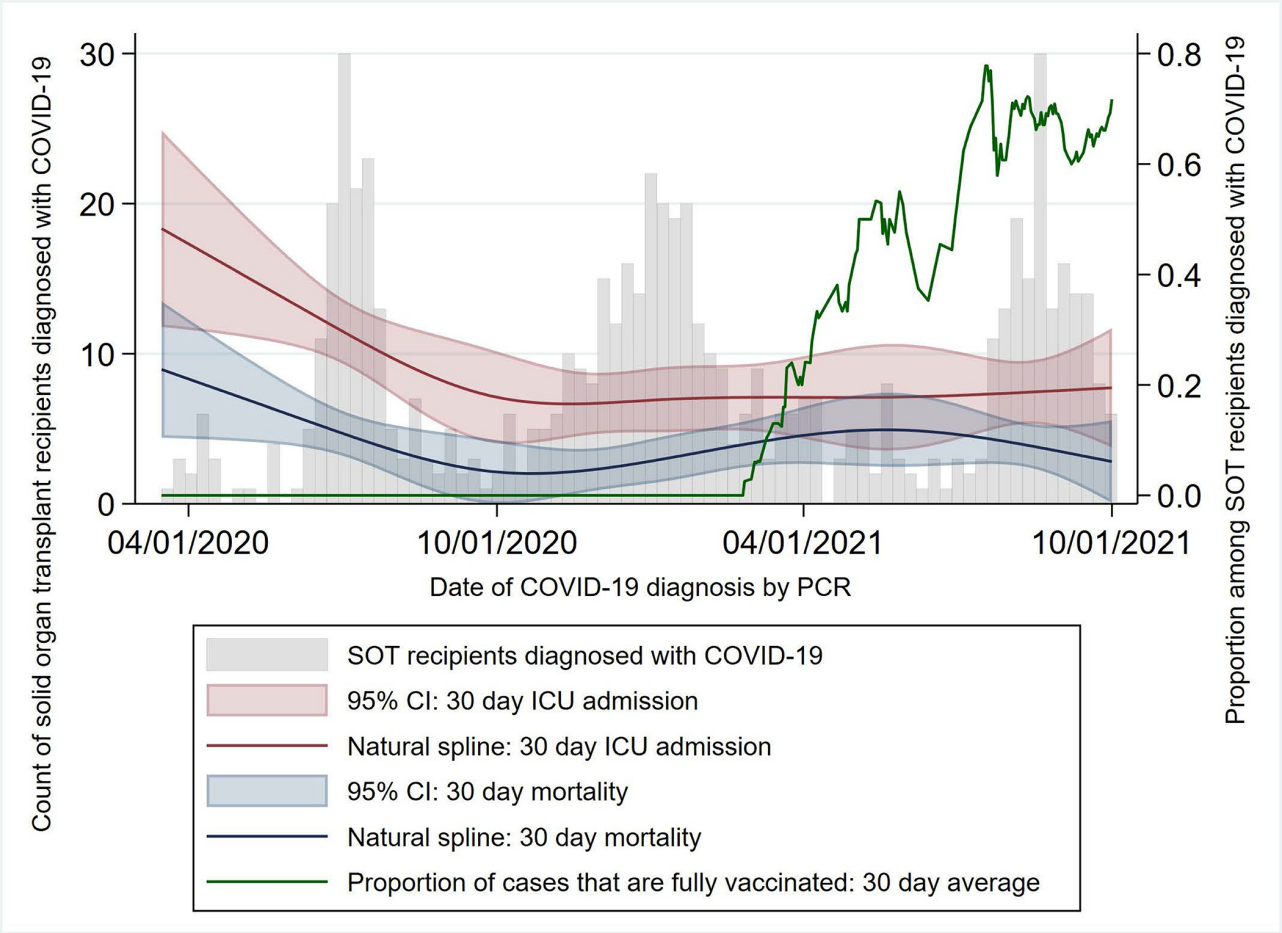
COVID-19 in solid organ transplant recipients over time. SOT: Solid organ transplant; ICU: Intensive care unit; CI: Confidence interval.

COVID-19 incidence among SOT recipients followed local epidemiologic trends, displaying four distinct peaks in April 2020, June 2020, January 2021, and August 2021 [28] (Fig 1). Following the initial peak, during which testing and treatment policies varied widely [21,29], 30 day mortality and 30 day ICU admission rates remained relatively stable, even as the proportion of patients diagnosed with COVID-19 after completing a vaccination course increased. In total, 300 (46%) SOT recipients were diagnosed with COVID-19 in 2020, before vaccinations were available, while 205 (32%) were diagnosed in 2021, but were not fully vaccinated at time of diagnosis. Within the latter category, 31/205 (15%) had received at least one vaccine dose but were not considered fully vaccinated under the CDC definition. Among the remaining patients, 90 (14%) completed their vaccination course within the 179 days prior to their diagnosis, while 51 (8%) were vaccinated 180 days or more before their diagnosis; median time from vaccine course completion to COVID-19 diagnosis was 160 days (IQR 108–190 days). The study cohort did not contain any transplant patients received the viral vector JNJ-78436735 (Johnson & Johnson) vaccine. Among all study patients, 154 (24%) received azithromycin, 130 (20%) received methylprednisolone, and 225 (35%) received dexamethasone within their diagnostic encounter. In addition, 136 (21%) patients received monoclonal antibody treatment for COVID-19. Of 473 patients hospitalized within 30 days of COVID-19 diagnosis, 231 (49%) received remdesivir for COVID-19 treatment.

In total, of 646 SOT recipients under immunosuppressant regimens who were diagnosed with COVID-19 between March 1, 2020 and October 1, 2021, 99 (15.3%) expired before December 1, 2021, and 70 (79%) of those had COVID-19 listed as the primary cause of death with 66/70 (94.3%) patients died within 60 days of diagnosis. Median survival time for all expired patients was 28 days from COVID-19 diagnosis (IQR 15–63 days, range 1–585 days), while median survival time for patients who died from COVID-19 was 21 days (IQR 13–29 days, range 1–82 days) (Fig 1). The primary outcome of COVID-19 mortality within 60 days of diagnosis was used in survival analyses. Of the 66 patients who died from COVID-19 within 60 days of diagnosis, 57 (86%) were unvaccinated, 4 (6%) had been vaccinated less than 180 days before being diagnosed, and 5 (8%) had been vaccinated 180 days or more before being diagnosed. In multivariable Cox proportional hazard models, increasing age was a risk factor for 60 day COVID-19 mortality with hazard ratio (HR): 1.04 (5% CI: 1.01–1.16); p-value: 0.001, while vaccination within 180 days of diagnosis was protective, compared to unvaccinated patients diagnosed in 2021, HR: 0.31 (95% CI 0.11–0.90); p-value: 0.03. Notably, vaccination at 180 days or longer was not protective against COVID-19 mortality, HR 0.70 (95% CI 0.26–1.85); p-value: 0.47; furthermore, mortality risk did not vary significantly between SOT recipients diagnosed in 2020 and unvaccinated SOT recipients diagnosed in 2021 (Table 2).

**Table 2:** 60-day COVID-19 mortality among solid organ transplant recipients.

	Univariable		Multivariable	
Cox Proportional Hazard model	(N = 646)		(N = 646)	
Event: COVID-19 mortality, n = 66	*HR (95% CI)*	*p-value*	*HR (95% CI)*	*p-value*
Age at encounter (years)	1.03 (1.01, 1.05)	0.01	1.04 (1.01, 1.06)	0.001
Gender				
Female	(reference)		(reference)	
Male	0.85 (0.53, 1.38)	0.52	0.79 (0.49, 1.29)	0.36
Race/Ethnicity				
NH White	(reference)			
NH Black	1.31 (0.72, 2.37)	0.37		
NH Asian	0.70 (0.16, 2.9)	0.62		
NH Other Race	0.98 (0.13, 7.26)	0.99		
Hispanic or Latino	1.02 (0.56, 1.86)	0.96		
Body Mass Index	1.02 (0.99, 1.06)	0.22		
Charlson Comorbidity Index Score	1.04 (0.99, 1.10)	0.13		
Transplanted organ				
Multi-organ	0.96 (0.23, 3.97)	0.95	1.04 (0.25, 4.37)	0.96
Heart	0.58 (0.21, 1.62)	0.29	0.46 (0.16, 1.30)	0.14
Kidney	(reference)		(reference)	
Liver	0.84 (0.42, 1.67)	0.61	0.70 (0.34, 1.40)	0.31
Lung	1.04 (0.48, 2.22)	0.92	0.90 (0.42, 1.93)	0.78
Kidney/Pancreas	0.85 (0.26, 2.75)	0.79	1.09 (0.33, 3.58)	0.89
Time from transplant to COVID-19 diagnosis (years)	1.02 (0.98, 1.06)	0.27		
Vaccination status				
Unvaccinated, diagnosed in 2020	0.93 (0.55, 1.57)	0.78	0.92 (0.54, 1.55)	0.75
Unvaccinated, diagnosed in 2021	(reference)		(reference)	
Diagnosed <180 days after vaccination	0.36 (0.13, 1.05)	0.06	0.31 (0.11, 0.90)	0.03
Diagnosed 180+ days after vaccination	0.84 (0.32, 2.20)	0.72	0.70 (0.26, 1.85)	0.47

Model notes: Includes all SOT recipients receiving immunosuppressant treatments at time of COVID-19 diagnosis. Outcome: Mortality from COVID-19 within 60 days of COVID-19 diagnosis; HR, hazard ratio; CI, confidence interval.

Of 646 SOT recipients, 136 (21%) were admitted to the ICU within 30 days of COVID-19 diagnosis; 117/136 (86%) of patients admitted to the ICU were unvaccinated. In multivariable logistic regression analysis, an increasing Charlson Comorbidity Index score was a risk factor for 30-day ICU admission, odds ratio (OR): 1.09 (95% CI 1.04–1.15); p-value: <0.001, while vaccination within 180 days of diagnosis was again protective, compared to unvaccinated patients diagnosed in 2021, OR: 0.45 (0.21–0.95); p-value: 0.04 (Table 3).

**Table 3:** Risk of ICU admission among solid organ transplant recipients diagnosed with COVID-19.

	Univariable		Multivariable	
Logistic regression model	(N = 646)		(N = 626)	
Event: ICU admission, n = 136	*OR (95% CI)*	*p-value*	*OR (95% CI)*	*p-value*
Age at encounter (years)	1.01 (1.00, 1.03)	0.051	--	--
Gender				
Female	(reference)		--	--
Male	1.11 (0.76, 1.63)	0.58	--	--
Race/Ethnicity				
NH White	(reference)		--	--
NH Black	1.13 (0.70, 1.81)	0.62	--	--
NH Asian	0.41 (0.12, 1.39)	0.15	--	--
NH Other Race	0.36 (0.05, 2.91)	0.34	--	--
Hispanic or Latino	0.95 (0.60, 1.51)	0.84	--	--
Body Mass Index	1.02 (0.99, 1.05)	0.27	1.02 (0.99, 1.05)	0.18
Charlson Comorbidity Index Score	1.08 (1.04, 1.13)	<0.001	1.09 (1.04, 1.15)	<0.001
Transplanted organ				
Multi-organ	1.38 (0.48, 3.95)	0.55	1.34 (0.44, 4.10)	0.61
Heart	0.73 (0.35, 1.50)	0.39	0.53 (0.25, 1.12)	0.1
Kidney	(reference)		(reference)	
Liver	1.10 (0.66, 1.86)	0.71	0.72 (0.40, 1.29)	0.27
Lung	1.44 (0.80, 2.59)	0.22	1.16 (0.62, 2.16)	0.64
Kidney/Pancreas	0.74 (0.28, 2.00)	0.56	0.76 (0.28, 2.10)	0.6
Time from transplant to COVID-19 diagnosis (years)	1.00 (0.97, 1.04)	0.85		
Vaccination status				
Unvaccinated, diagnosed in 2020	1.29 (0.84, 1.99)	0.24	1.30 (0.84, 2.01)	0.25
Unvaccinated, diagnosed in 2021	(reference)		(reference)	
Diagnosed <180 days after vaccination	0.49 (0.23, 1.02)	0.06	0.45 (0.21, 0.95)	0.04
Diagnosed 180+ days after vaccination	0.83 (0.38, 1.84)	0.65	0.68 (0.30, 1.53)	0.35

Model notes: Includes all SOT recipients receiving immunosuppressant treatments at time of COVID-19 diagnosis. Outcome: ICU admission within 30 days of COVID-19 diagnosis; OR, *odds ratio*; CI: *Confidence interval;* ICU: *Intensive care unit*.

Patient characteristics in the PS matched cohort was present in Table 4, which showed all evaluated criteria were balanced between patients with and without vaccination.

**Table 4:** Demographics and clinical characteristics of solid organ transplant recipients diagnosed with COVID-19 by vaccination status, propensity score matched cohort.

	Total	Not vaccinated	Vaccinated	Standardized	p-value
Characteristics	N = 282	N = 141	N = 141	bias %	
**Demographics**	* *	* *	* *	* *	
Age at encounter (years), median (IQR)	60.0 (52.0, 67.0)	59.0 (51.0, 66.0)	61.0 (52.0, 68.0)	7.5	0.53
Gender					
Female	127 (45.0)	62 (44.0)	65 (46.1)	4.3	0.72
Male	155 (55.0)	79 (56.0)	76 (53.9)	-4.3	0.72
Race/Ethnicity					
Non-Hispanic White	121 (42.9)	65 (46.1)	56 (39.7)	-12.9	0.28
Non-Hispanic Black	69 (24.5)	29 (20.6)	40 (28.4)	18.2	0.13
Non-Hispanic Asian	15 (5.3)	6 (4.3)	9 (6.4)	9.5	0.43
Hispanic or Latino	77 (27.3)	41 (29.1)	36 (25.5)	-7.9	0.51
**Clinical characteristics**	** **	** **	** **	** **	** **
Body mass index, median (IQR)	28.3 (24.3, 32.8)	28.3 (24.3, 32.5)	27.8 (24.4, 33.0)		0.65
Charlson Comorbidity Index Score, median (IQR)	8.5 (6.0, 11.0)	9.0 (6.0, 11.0)	8.0 (6.0, 11.0)	0.7	0.95
**Medical history **					
Chronic obstructive pulmonary disease	123 (43.6)	62 (44.0)	61 (43.3)	-1.4	0.91
Tuberculosis	4 (1.4)	2 (1.4)	2 (1.4)	0.0	1.00
Dementia	103 (36.5)	46 (32.6)	57 (40.4)	16.2	0.18
Myocardial Infarction	137 (48.6)	68 (48.2)	69 (48.9)	1.4	0.91
Peripheral vascular disease	155 (55.0)	81 (57.4)	74 (52.5)	-10.0	0.40
Congestive heart failure	118 (41.8)	64 (45.4)	54 (38.3)	-14.4	0.23
Cerebrovascular disease	29 (10.3)	13 (9.2)	16 (11.3)	7.0	0.56
Diabetes	118 (41.8)	58 (41.1)	60 (42.6)	2.9	0.81
Peptic ulcer disease	211 (74.8)	108 (76.6)	103 (73.0)	-8.1	0.49
Liver disease	6 (2.1)	5 (3.5)	1 (0.7)	-19.7	0.10
Renal disease	272 (96.5)	137 (97.2)	135 (95.7)	-7.6	0.52
Hemiplegia	65 (23.0)	31 (22.0)	34 (24.1)	5.0	0.67
Cancer	26 (9.2)	11 (7.8)	15 (10.6)	9.8	0.41
HIV/AIDS	2 (0.7)	1 (0.7)	1 (0.7)	0.0	1.00
**Transplant characteristics**	** **	** **	** **	** **	** **
Transplanted organ					
Multi-organ	9 (3.2)	3 (2.1)	6 (4.3)	12.1	0.31
Heart	26 (9.2)	16 (11.3)	10 (7.1)	-14.7	0.22
Kidney	147 (52.1)	66 (46.8)	81 (57.4)	21.3	0.07
Liver	51 (18.1)	29 (20.6)	22 (15.6)	-12.9	0.28
Lung	37 (13.1)	20 (14.2)	17 (12.1)	-6.3	0.60
Kidney/Pancreas	12 (4.3)	7 (5.0)	5 (3.5)	-7.0	0.56
Year of most recent transplant					
<2015	95 (33.7)	41 (29.1)	54 (38.3)	19.5	0.10
2015	24 (8.5)	15 (10.6)	9 (6.4)	-15.2	0.20
2016	19 (6.7)	10 (7.1)	9 (6.4)	-2.8	0.81
2017	27 (9.6)	15 (10.6)	12 (8.5)	-7.2	0.55
2018	20 (7.1)	12 (8.5)	8 (5.7)	-11.0	0.36
2019	35 (12.4)	24 (17.0)	11 (7.8)	-28.1	0.02
2020	49 (17.4)	19 (13.5)	30 (21.3)	20.6	0.08
2021	13 (4.6)	5 (3.5)	8 (5.7)	10.1	0.40
Time from transplant to COVID-19 diagnosis (years), median IQR	4.4 (1.4, 8.5)	3.8 (1.4, 6.9)	4.8 (1.3, 9.5)	12.6	0.29
Immunosuppressant treatments at COVID-19 diagnosis					
Antithymocyte globulin	2 (0.7)	1 (0.7)	1 (0.7)	0.0	1.00
Tacrolimus	250 (88.7)	127 (90.1)	123 (87.2)	-8.9	0.45
Cyclosporine	14 (5.0)	5 (3.5)	9 (6.4)	13.0	0.27
Mycophenolate	238 (84.4)	121 (85.8)	117 (83.0)	-7.8	0.51
Azathioprine	1 (0.4)	0 (0.0)	1 (0.7)	11.9	0.32
Sirolimus	25 (8.9)	13 (9.2)	12 (8.5)	-2.5	0.84
Everolimus	9 (3.2)	4 (2.8)	5 (3.5)	4.0	0.74
Belatacept	4 (1.4)	2 (1.4)	2 (1.4)	0.0	1.00
Prednisone	254 (90.1)	127 (90.1)	127 (90.1)	0.0	1.00
COVID-19 treatments administered at diagnostic encounter					
Azithromycin	55 (19.5)	30 (21.3)	25 (17.7)	-8.9	0.45
Methylprednisolone	63 (22.3)	35 (24.8)	28 (19.9)	-11.9	0.32
Ribavirin	0 (0.0)	0 (0.0)	0 (0.0)	.	.
Tocilizumab	3 (1.1)	2 (1.4)	1 (0.7)	-6.9	0.56
Dexamethasone	98 (34.8)	49 (34.8)	49 (34.8)	0.0	1.00
Remdesivir	127 (45.0)	65 (46.1)	62 (44.0)	-4.3	0.72
Monoclonal antibodies	122 (43.3)	62 (44.0)	60 (42.6)	-2.9	0.81

IQR: *Interquartile range*; ICU: *Intensive care unit;* Differences between exposure groups compared using chi-square or Fisher’s exact tests for categorical variables and Kruskal Wallis test for continuous variables.

The Cox regression model run on the PS matched cohort confirmed the association between the vaccination and lower mortality within 60 days with a multivariable HR of 0.28 (95% CI 0.08, 0.94), p = 0.04 (Table 5). The multivariable logistic regression model run on the PS matched cohort also found patients who received vaccination with 180 days from diagnosis had lower odds of ICU admission, OR 0.21 (95% CI 0.08, 0.51), p = 0.001 (Table 6).

**Table 5:** 60-day COVID-19 mortality among solid organ transplant recipients, in propensity score matched cohort (N = 282).

	Multivariable	
Characteristics	HR (95% CI)	p-value
Age at encounter (years)	1.04 (1.00, 1.08)	0.06
Vaccination status		
Unvaccinated, diagnosed in 2020	0.55 (0.20, 1.51)	0.24
Unvaccinated, diagnosed in 2021	REF	
Diagnosed <180 days after vaccination	0.28 (0.08, 0.94)	0.04
Diagnosed 180+ days after vaccination	0.77 (0.25, 2.37)	0.65
Monoclonal antibodies	0.05 (0.01, 0.36)	0.003

HR, hazard ratio; CI, confidence interval; REF, reference group.

**Table 6:** Risk of ICU admission among solid organ transplant recipients diagnosed with COVID-19, in the propensity score matched cohort (N = 282).

	Multivariable	
Characteristics	OR (95% CI)	p-value
Age at encounter (years)	0.99 (0.96, 1.02)	0.41
Male gender	1.30 (0.67, 2.50)	0.44
Charlson Comorbidity Index Score	1.16 (1.06, 1.28)	0.002
Transplanted organ		
Multi-organ	1.10 (0.17, 6.92)	0.92
Heart	0.69 (0.22, 2.17)	0.53
Kidney	REF	
Liver	1.02 (0.41, 2.51)	0.97
Lung	1.63 (0.58, 4.55)	0.35
Kidney/Pancreas	1.50 (0.30, 7.90)	0.65
Vaccination status		
Unvaccinated, diagnosed in 2020	0.58 (0.25, 1.36)	0.21
Unvaccinated, diagnosed in 2021	REF	
Diagnosed <180 days after vaccination	0.21 (0.08, 0.51)	0.001
Diagnosed 180+ days after vaccination	0.40 (0.15, 1.06)	0.07
Monoclonal antibodies	0.19 (0.08, 0.41)	<0.001

OR, odds ratio; CI, confidence interval; REF, reference group.

## Discussion

Our findings add to the growing body of literature demonstrating that, in spite of chronic immunosuppression and ostensibly a poor humoral response to the mRNA vaccines, mortality may be reduced among fully vaccinated solid organ transplant recipients who are exposed to COVID-19, compared to partially vaccinated or unvaccinated patients [30–32]. The presence of neutralizing antibodies could plausibly contribute to the observed reduction in COVID-19 case fatality among fully vaccinated SOT patients. However, new research indicates the production of neutralizing antibodies may also be hindered by the immunosuppressive drugs used in SOT recipients [33,34]. Therefore, future research is needed to identify additional mechanisms, such as a potentially protective role of cellular (T-cell) immunity, to explain the protective effect of vaccination without accompanying immunogenicity [35]. Additionally, further investigation should determine the effect of novel SARS-CoV-2 variants on disease dynamics within immunosuppressed populations. We also demonstrated that vaccination may offer waning protection from poor COVID-19 outcomes over time in an immunosuppressed population; SOT recipients in our cohort diagnosed with COVID-19 six months or more after being vaccinated were at similar risk for both COVID-19 mortality and ICU admission as patients who were never vaccinated. This study was restricted to patients without a known history of previous COVID-19 infection, and further research is needed to determine how vaccine immunogenicity may vary among immunosuppressed survivors of COVID-19.

Our study has several limitations. First, given this is a retrospective study, some clinical parameters were not collected in our dataset. For example, time from the first symptoms to diagnosis of COVID-19 was not available which may affect the outcome as we could not determine if the vaccinated patients were more likely to seek medical care earlier than unvaccinated patients. However, the analysis on the PS matched cohort confirmed that vaccination within 180 days of diagnosis was associated with a lower mortality within 60 days of diagnosis or ICU admission, independently with the treatment of remdesivir or monoclonal antibodies. Second, our data were obtained from one hospital system, which might not be generalized to other populations. Third, data of patients who had at least three doses of vaccines were not available in our patients for the time being. Of note, while patients in our study were considered as “fully vaccinated” with 2 vaccine doses as per the CDC definition, SOT recipients ages 5 years and older are recommended by the American Society of Transplantation to receive an initial three-dose series of mRNA vaccine followed by one or two booster doses if ages 12 or older [36].” Fourth, although we tried our best to rule out patients with a known history of previous COVID-19 infection, we may have missed some previous infections given lack of the confirmation of anti-N SARS-CoV-2 antibodies. Our analysis may also underestimate the patient outcome as we could not rule out completely the possibility that the patient sought further care at a different institution after their initial encounter at the Houston Methodist Hospital System. Finally, our findings may need to be considered with a gap between the data collected during the pandemic period at a single center and the current COVID-19 situation, especially with the fast-pace of evolving new SARS-CoV-2 variants and the change in the vaccine recommendations for SOT recipients from 2 doses to a primary 3-dose course followed by boosters.

Despite the limitations, our study has notable strengths. Our study is strengthened by a robust sample size of SOT recipients with COVID-19, a diverse, heterogeneous population, precise EHR-derived clinical information, and significant longitudinal follow-up of COVID-19 patients. While overall COVID-19 case fatality rates have decreased over time following the introduction of more effective treatment strategies [10], among our population of immunosuppressed SOT recipients, COVID-19 mortality and ICU admission rates did not vary significantly after the initial peak in April 2020. This investigation was a single-center, registry-based chart-review, so while patients could have experienced un-observed or un-documented outcomes at other institutions, all SOT recipients routinely received SARS-CoV-2 testing for surveillance purposes during the study period, regardless of symptoms. The observed difference between the fully vaccinated and unvaccinated patients can inform clinical practice and warrants additional studies as large-scale vaccination efforts continue. Moreover, the observed loss of protection within months of vaccination lends crucial evidence to discussions of booster recommendations in immunologically at-risk populations. Future investigations may prioritize longitudinal follow-up of vaccinated patients to determine incidence of additional markers of morbidity and poor health outcomes over time. This study demonstrated an association between mRNA vaccination and reduced mortality in solid organ transplant recipients; these results can contribute to the development of comprehensive vaccination programs targeting high-risk, immunosuppressed populations.

## Abbreviations

SOT: *sold organ transplant*
IQR: *Interquartile range*
HR: *hazard ratio*
OR: *odds ratio*
CI: *confidence interval*
ICU: *Intensive care unit*
